# Development of the Global Disability Scale (Glo.Di.S): preliminary results

**DOI:** 10.1186/1744-859X-11-14

**Published:** 2012-05-17

**Authors:** Konstantinos N Fountoulakis, Eirini Lekka, Evangelia Kouidi, Ioanna Chouvarda, Asterios Deligiannis, Nickolaos Maglaveras

**Affiliations:** 1 Department of Psychiatry, School of Medicine, Aristotle University of Thessaloniki, Thessaloniki, Pylaia 55535, Greece; 2 Lab of Medical Informatics, School of Medicine, Aristotle University of Thessaloniki, Thessaloniki, Greece; 3 Laboratory of Sports Medicine, Aristotle University of Thessaloniki, Thessaloniki, Greece

**Keywords:** Disability, Burden, Functioning, ICF, Development, Impairment

## Abstract

**Background:**

The assessment of functioning and disability is an important part of the clinical evaluation, since it measures disease burden and reflects the effectiveness of therapeutic planning and interventions. The aim of the current study was to develop such a self-report instrument on the basis of a review of the literature, and compatible with the WHO approach.

**Material and methods:**

The review of the literature led to the development of the Global Disability Scale (Glo.Di.S) with 25 items assessing different aspects of disability. The study sample included 728 persons from vulnerable populations (homeless, jobless, very low income, single parent families etc.; (29.12% males and 70.88% females; aged 55.96 ± 15.22 years). The protocol included also the STAI and the CES-D. The statistical analysis included factor analysis item analysis and ANCOVA.

**Results:**

The factor analysis revealed the presence of 4 factors explaining 71% of total variance (Everyday functioning, Social and interpersonal functioning, Severity and Mental disability). Chronbach’s alpha for the whole scale was 0.95 and for subscales were 0.74–0.94.

**Discussion:**

The results of the current study suggest that the Glo.Di.S. has the potential to serve as a reliable and valid tool for assessing functioning and disability. Further research is needed to prove that it could be useful across countries, populations and diseases, and whether it provides data that are culturally meaningful and comparable. It can be used in surveys and in clinical research settings and it can generate information of use in evaluating health needs and the effectiveness of interventions to reduce disability and improve health.

## Background

During the last few decades, the assessment of functioning and disability became important and part of the clinical evaluation, since it shows the ability of an individual to function in general areas of life. Along with mortality, morbidity and other rates reflecting a population’s health status, disability is important in order to measure disease burden and to evaluate the effectiveness of therapeutic planning and interventions. The description of clinical symptoms and their response to treatment alone does not seem to be sufficient for the comprehensive understanding of the disease and the needs of the patients. The addition of the assessment of disability enhances patient management, intervention design and the reporting of health [1].

The literature strongly suggests that symptom severity does not always correlate strongly with disability and only specific domains of the clinical picture seem to be responsible for the observed functional impairment. It is also reported that only a small proportion of the observed variability for disability is explained by any combination of clinical symptoms [2-8].

However, in spite of recommendations, this assessment has not yet become part of routine every day clinical practice. This is partially because defining and measuring disability has always been a challenge. In clinical practice it is a challenge both in terms of skills and time consuming to assess the individual patients’ broad spectrum of problems precisely, and areas like life satisfaction, quality of life or sexual functioning are poorly covered by interviewers [9].

In order to address this challenge, several instruments have been developed so far and a huge number of concepts and definitions have been developed and operationalized [10].

The World Health Organization (WHO) established the International Classification of Functioning, Disability and Health (ICF) [11]. The joint use of the ICD-10 [12] and the ICF serves this purpose [1]. The WHO also developed a standard instrument the World Health Organization Disability Assessment Schedule (WHODAS) [13]. The WHODAS captures an individual’s level of functioning in six major life domains: (i) cognition (understanding and communication); (ii) mobility (ability to move and get around); (iii) self-care (ability to attend to personal hygiene, dressing and eating, and to live alone); (iv) getting along (ability to interact with other people); (v) life activities (ability to carry out responsibilities at home, work and school); (vi) participation in society (ability to engage in community, civil and recreational activities).

It is true that existing disability measures in psychiatry are either comprehensive but lengthy, or too short and uni-dimentional. There is a need for a user friendly, relatively short but comprehensive, simple, cost-effective, and sensitive measure of disability and functional impairment in clinical practice. The aim of the current study was to develop such a self-report instrument on the basis of a systematic review of the literature, and compatible with the WHO approach.

## Material and methods

### Development of the scale

The first step was to review the literature and indentify scales assessing disability. The MEDLINE search returned 104 articles relevant for the current study. The review led to the development of 25 items assessing different aspects of disability. They comprised the Global Disability Scale (Glo.Di.S). It is essential to report that the WHODAS [13] and its content were extremely influential in the development of this scale.

The scoring method was developed after consensus by three of the researchers (KNF, AD and NM) and it was assumed that a mentally and physically healthy person aged below 50 would receive a score close to zero in all items of the scale. The scoring method included four options as response to each item (0 = not at all, 1 = a little, 2 = moderately, 3 = severely and 4 = very severe or complete disability). The Glo.Di.S scale and its subscales are shown in the Additional file 1.

The gathering of the data was done by four trained psychologists with the use of an internet-based electronic platform. In this frame, although he Glo.Di.S was developed as a self-report tool, for the particular study the questions were read by the interviewer and the subject was choosing the right answer as it is was a strict structured interview with no input at all from the side of the interviewer.

The study was approved by the Ethics Committee of the Aristotle University Medical School. All subjects gave their informed consent. All procedures were approved by the National Authority for the protection of personal and private data.

### Study population

In the frame of a study on vulnerable populations (homeless, jobless, very low income, single parent families etc.) sponsored by the Municipality of Thessaloniki Greece, these items were applied to 728 subjects from this particular population (29.12% males and 70.88% females) aged 55.96 ± 15.22 (range 19–94 years).

### Psychometric assessment

Apart from demographic variables, the protocol included the registration of health status with codes for the major disease categories according to the ICD-10 [12] and the World Health Organization. It also registered height and weight, details about current and past occupational status and alcohol and drug use and abuse. Anxiety was assessed with the STAI [14] and depression with the CES-D [15]. The test-retest reliability was not studied. It is also important to note that the collection of the data has been completed by 2009, that is, essentially before the current economic crisis really began.

### Statistical analysis

The analysis included the development of descriptive statistic tables for the study sample, and specifically frequency tables for each reply in each item of the new scale.

The statistical analysis included factor analysis with Varimax normalized rotation, item analysis and the calculation of Cronbach’s alpha, and the calculation of Pearson Product moment correlation coefficient between the new scale and age, BMI, STAI-S, STAI-T and CES-D. The Analysis of Covariate (ANCOVA) was also performed with total Glo.Di.S. score and subscales scores as dependent variables, sex as categorical predictor and age, BMI, STAI-S and T and CES-D scores as continues predictors.

## Results

The frequencies and mean scores for each of the scale items are shown in Table 1. The factor analysis revealed the presence of 4 factors explaining 71% of total variance. The first one included items 1–10, 16 and 21, the second included 17–20 and 22, the third included 1, 11–13, 21 and 23–25 while the fourth included 8, 14–16, 18 and 21. Items 1, 8, 16 and 18 loaded almost equally in two factors, while item 21 (sexual life) loaded in three factors (Table 2). Secondary factor analysis revealed a single secondary factor structure.

**Table 1:** Frequency of responses and mean score in the various items and subscales of the Glo.Di.S

**Item no.**	**Item**	**Frequency of responses**					**Score**	
		**0**	**1**	**2**	**3**	**4**	**mean**	**SD**
1	Stand up from sitting	70.76	10.76	9.38	6.34	2.76	0.60	1.07
2	Dress by yourself	91.60	2.20	2.89	2.07	1.24	0.19	0.70
3	Eat by yourself	96.69	1.24	0.41	0.97	0.69	0.08	0.47
4	Move around your house by yourself	87.72	4.97	2.76	3.31	1.24	0.25	0.77
5	Take a bath or a shower by yourself	87.05	3.72	3.03	4.27	1.93	0.30	0.87
6	Carryout the most important works in the house	75.59	9.52	6.07	6.21	2.62	0.51	1.03
7	Complete all the works of the house	74.90	8.83	6.90	6.62	2.76	0.54	1.06
8	Carry on your everyday work	77.76	5.94	5.25	8.29	2.76	0.52	1.09
9	Stay alone for a few days	78.65	4.82	5.10	7.44	3.99	0.53	1.13
10	Get out of the house for a walk, shopping etc.	81.40	5.65	4.41	5.79	2.75	0.43	1.01
11	Deal with various obstacles or physically demanding situations (stairs, taking the bus etc.)	53.52	14.48	9.24	17.66	5.10	1.06	1.34
12	Stand for some time (15–30 minutes)	58.26	10.19	8.40	16.12	7.02	1.03	1.39
13	Walk for a distance of around a kilometer	58.34	9.38	7.72	16.14	8.41	1.07	1.44
14	Concentrate on something for 5–10 minutes (Newspaper, TV, cooking)	88.57	4.55	3.44	3.17	0.28	0.22	0.68
15	Learn something new (how to go to a new place, a new recipe etc)	83.03	4.97	3.59	6.76	1.66	0.39	0.96
16	Participate in the activities of the community (e.g. religious, celebrations etc.)	80.80	4.42	5.52	7.73	1.52	0.45	1.00
17	Handle your relationships with people close to you (friends, relatives etc.)	84.00	4.69	6.07	4.55	0.69	0.33	0.84
18	Socialize with people you don’t know	86.60	4.56	5.11	3.59	0.14	0.26	0.73
19	Keep a friendship	87.02	4.42	4.56	4.01	0.00	0.26	0.72
20	Make new friends	81.24	4.83	5.66	8.00	0.28	0.41	0.93
21	Have sexual life	75.21	2.62	3.99	12.12	6.06	0.71	1.32
22	Live with dignity because of your problem	73.76	6.49	5.80	12.85	1.10	0.61	1.12
23	How much time you dedicated to your health issues and their consequences?	60.33	11.43	9.78	17.36	1.10	0.87	1.22
24	How emotionally distressed are you because of your health?	56.14	7.59	10.76	22.07	3.45	1.09	1.36
25	How much of an economic burden to you and your family is your health?	69.15	7.85	8.82	12.40	1.79	0.70	1.16
	Everyday functioning							
	Social and interpersonal functioning							
	Severity							
	Mental disability							
	Glo.Di.S total score							

**Table 2:** Factor analysis

**Glo.Di.S item**	**Factor 1**	**Factor 2**	**Factor 3**	**Factor 4**
Stand up from sitting	***0.57***	0.11	***0.63***	0.23
Dress by yourself	***0.84***	0.14	0.25	0.10
Eat by yourself	***0.73***	0.31	-0.05	-0.15
Move around your house by yourself	***0.81***	0.12	0.32	0.21
Take a bath or a shower by yourself	***0.80***	0.13	0.31	0.25
Carryout the most important works in the house	***0.70***	0.14	0.40	0.45
Complete all the works of the house	***0.65***	0.11	0.45	0.44
Carry on your everyday work	***0.54***	0.28	0.37	
Stay alone for a few days	***0.54***	0.21	0.31	0.22
Get out of the house for a walk, shopping etc.	***0.68***	0.08	0.47	0.37
Deal with various obstacles or physically demanding situations (stairs, taking the bus etc.)	0.29	0.14	***0.83***	0.08
Stand for some time (15–30 minutes)	0.28	0.15	***0.85***	0.13
Walk for a distance of around a kilometer	0.27	0.13	***0.85***	0.13
Concentrate on something for 5–10 minutes (Newspaper, TV, cooking)	0.09	0.27	0.17	***0.73***
Learn something new (how to go to a new place, a new recipe etc)	0.26	0.33	0.17	***0.64***
Participate in the activities of the community (e.g. religious, celebrations etc.)	***0.43***	0.34	0.39	***0.54***
Handle your relationships with people close to you (friends, relatives etc.)	0.18	***0.64***	0.20	0.21
Socialize with people you don’t know	0.26	***0.66***	0.10	***0.51***
Keep a friendship	0.20	***0.82***	0.09	0.25
Make new friends	0.05	***0.84***	0.13	0.23
Have sexual life	***0.44***	0.24	***0.37***	***0.39***
Live with dignity because of your problem	0.18	***0.48***	0.38	-0.11
How much time you dedicated to your health issues and their consequences?	0.20	0.25	***0.69***	0.38
How emotionally distressed are you because of your health?	0.13	0.33	***0.66***	0.39
How much of an economic burden to you and your family is your health?	0.34	0.03	***0.55***	0.42
Explained variance	5.85	3.36	5.38	3.35
Proportion of variance explained	23%	13%	22%	13%
Total variance explained				71%

These four factors correspond to four subscales. The first one is ‘everyday functioning’. The second corresponds to ‘Social and interpersonal functioning’, the third to ‘severity’ and the fourth to ‘mental disability’. The mean scores for each of the subscales as well as the mean total score are shown in Table 1.

Chronbach’s alpha for the whole scale was 0.95 and for subscales were 0.94, 0.74, 0.92 and 0.75 respectively.

The ANCOVA suggested no differences between males and females. However age, BMI and CES-D score were significantly correlated with disability. The correlation matrix between the new scale and age, BMI, STAI-S, STAI-T and CES-D is shown in Table 3.

**Table 3:** The correlation matrix between the new scale and age, BMI, STAI-S, STAI-T and CES-D

	**Total Glo.Di.S score**	**Everyday functioning**	**Social and interpersonal functioning**	**Severity**	**Mental disability**
**age**	***0.29***	***0.27***	0.07	***0.33***	***0.29***
**BMI**	***0.13***	***0.10***	0.05	***0.16***	***0.09***
**STAI-T**	***0.18***	***0.11***	***0.28***	***0.17***	***0.16***
**STAI-S**	***0.18***	***0.11***	***0.26***	***0.16***	***0.14***
**CES-D**	***0.34***	***0.25***	***0.40***	***0.33***	***0.29***

All values significant at p < 0.05 are marked in bold italics underlined.

## Discussion

The current paper reports the preliminary results from the development of the Global Disability Scale (Glo.Di.S). This scale is a 25-items user friendly self report instrument which assesses disability in accord with the ICF approach of the WHO. It manifests high internal reliability (0.95) and consists of four subscales (Everyday functioning, Social and interpersonal functioning, Severity and Mental disability).

In comparison, the WHODAS-II has a mean Cronbach’s alpha equal to 0.65–0.98 [16-25], test-retest coefficients from 0.71–0.96 [19,23,25,26] and inter-rater reliability for individual subscales from 0.64–0.94 [20,21,25]. The short version might represent a single factor [27,28]. The large version consists probably of 6 subscales (understanding and communicating, getting around, self-care, getting along with people, life activities and participation in society) and a single superfactor (a two-factor solution is also possible) [19,24].

The screener part of the ICF Measure of Participation and ACTivities questionnaire (IMPACT-S) consists of 9 scales, reflecting the 9 activity and participation chapters of the ICF. Again the Chronbach’s alpha was satisfying for all 9 domains (0.75–0.89) and excellent for the total score (0.96). The test-retest reliability was good at item level (0.44–0.72), domain level (0.72–0.92) and total score (0.94) [29]. The Lam Employment Absence and Productivity Scale (LEAPS), which is a 10-item self-report questionnaire has a Cronbach’s alpha of 0.89 [30]. The widely used self-report Sheehan Disability Scale (SDS) has a single factor structure [31-34] and Chronbach’s alpha >0.70 [31-33,35]. The test-retest reliability was 0.73 [33].

The Glo.Di.S manifests psychometric properties similar to the WHODAS and maybe superior to the other scales in terms of internal consistency.

The above instruments have already been used in the study of disability across a number of sociodemographic variables and different diseases and disorders, including acne vulgaris with social phobia [36], bipolar disorder [37-39], undifferentiated peripheral inflammatory arthritis [40], adult-onset hearing loss [41], psychotic disorders [42-47], multiple sclerosis [47], depression and anxiety [7,46,48-60], injury [61-63], alcohol abuse [64], sex differences [53,65-67], arthritis [60], chronic spinal pain [60], high blood pressure [60], social anxiety [68], obsessive-compulsive disorder [69], on-pump coronary artery bypass [70], dementia [71], cancer with pain [72,73], obesity and type 2 diabetes mellitus [74], mental-physical disorders comorbidity [75,76], leprosy [77], ADHD [78,79], anxiety in children [80], Parkinson’s disease [81], macular degeneration [48], inflammatory arthritis [23], migraine [8] and systemic sclerosis [82,83].

All scales manifest a number of similar drawbacks. All of them fail to tackle specific aspects of disability. The WHODAS covers mainly the activities and participation domains of the ICF, so bodily impairments and environmental factors are not included. The SDS has similar limitations and is often inadequate to discern subtle, but important changes which may occur between measurements [84].

The Glo.Di.S. was developed in order to constitute a short, easy to use scale for the assessment of global disability and impairment. Its development aimed at constructing an instrument which would not mix functioning with symptomatology or subjective feelings and preferences. Instead the Glo.Di.S covers a broad area of disability and impairment and can be used in a variety of diseases including psychiatric disorders. Its structure will allow the comparison between disorders in an objective way.

## Conclusion

The results of the current study suggest that the Glo.Di.S. has the potential to serve as a reliable and valid tool for assessing functioning and disability. Further research is needed to prove that it could be useful across countries, populations and diseases, and whether it provides data that are culturally meaningful and comparable. It can be used in surveys and in clinical research settings and it can generate information of use in evaluating health needs and the effectiveness of interventions to reduce disability and improve health.

## Competing interests

The authors declare that they have no competing interests.

## Authors’ contributions

KNF and NM designed the protocol, analyzed the results, wrote the first draft and corrected all subsequent versions. EL, EK, IC and AD participated in the analysis and interpretation of results and corrected the drafts. All authors read and approved the final manuscript.

## Supplementary Material

Additional file 1 Global Disability Scale (Glo.Di.S). Click here for file
