# Supplementary material for: Development of the Global Disability Scale (Glo.Di.S): preliminary results
Source: Ann Gen Psychiatry. 2012 May 17;11:14. doi: 10.1186/1744-859X-11-14 (PMC3434028; doi:10.1186/1744-859X-11-14)
Supplement: Additional file 1 — Global Disability Scale (Glo.Di.S). [file 1744-859X-11-14-S1.doc]

**Additional file 1.** Global Disability Scale (Glo.Di.S)

|  | **During the last month how difficult was it for you by yourself to:** | **Not at all** | **A little** | **moderately** | **severely** | **Very severe or complete disability** |
| --- | --- | --- | --- | --- | --- | --- |
|  |  | **0** | **1** | **2** | **3** | **4** |
| 1 | Stand up from sitting | ❒ | ❒ | ❒ | ❒ | ❒ |
| 2 | Dress by yourself | ❒ | ❒ | ❒ | ❒ | ❒ |
| 3 | Eat by yourself | ❒ | ❒ | ❒ | ❒ | ❒ |
| 4 | Move around your house by yourself | ❒ | ❒ | ❒ | ❒ | ❒ |
| 5 | Take a bath or a shower by yourself | ❒ | ❒ | ❒ | ❒ | ❒ |
| 6 | Carryout the most important works in the house | ❒ | ❒ | ❒ | ❒ | ❒ |
| 7 | Complete all the works of the house | ❒ | ❒ | ❒ | ❒ | ❒ |
| 8 | Carry on your everyday work | ❒ | ❒ | ❒ | ❒ | ❒ |
| 9 | Stay alone for a few days | ❒ | ❒ | ❒ | ❒ | ❒ |
| 10 | Get out of the house for a walk, shopping etc. | ❒ | ❒ | ❒ | ❒ | ❒ |
| 11 | Deal with various obstacles or physically demanding situations (stairs, taking the bus etc.) | ❒ | ❒ | ❒ | ❒ | ❒ |
| 12 | Stand for some time (15-30 minutes) | ❒ | ❒ | ❒ | ❒ | ❒ |
| 13 | Walk for a distance of around a kilometer | ❒ | ❒ | ❒ | ❒ | ❒ |
| 14 | Concentrate on something for 5-10 minutes (Newspaper, TV, cooking) | ❒ | ❒ | ❒ | ❒ | ❒ |
| 15 | Learn something new (how to go to a new place, a new recipe etc) | ❒ | ❒ | ❒ | ❒ | ❒ |
| 16 | Participate in the activities of the community (e.g. religious, celebrations etc.) | ❒ | ❒ | ❒ | ❒ | ❒ |
| 17 | Handle your relationships with people close to you (friends, relatives etc.) | ❒ | ❒ | ❒ | ❒ | ❒ |
| 18 | Socialize with people you don’t know | ❒ | ❒ | ❒ | ❒ | ❒ |
| 19 | Keep a friendship | ❒ | ❒ | ❒ | ❒ | ❒ |
| 20 | Make new friends | ❒ | ❒ | ❒ | ❒ | ❒ |
| 21 | Have sexual life | ❒ | ❒ | ❒ | ❒ | ❒ |
| 22 | Live with dignity because of your problem | ❒ | ❒ | ❒ | ❒ | ❒ |
| 23 | How much time you dedicated to your health issues and their consequences? | ❒ | ❒ | ❒ | ❒ | ❒ |
| 24 | How emotionally distressed are you because of your health? | ❒ | ❒ | ❒ | ❒ | ❒ |
| 25 | How much of an economic burden to you and your family is your health? | ❒ | ❒ | ❒ | ❒ | ❒ |
